# Dental markers of poverty: Biocultural deliberations on oral health of the poor in mid‐nineteenth‐century Ireland

**DOI:** 10.1002/ajpa.23717

**Published:** 2018-10-03

**Authors:** Jonny Geber, Eileen Murphy

**Affiliations:** ^1^ Department of Anatomy University of Otago Dunedin New Zealand; ^2^ Archaeology and Palaeoecology, School of Natural and Built Environment, Queen's University Belfast Belfast Northern Ireland

**Keywords:** ante‐mortem tooth loss, dental caries, Great Irish Famine, periodontitis, smoking

## Abstract

**Objectives:**

Despite subsisting on a low‐cariogenic diet comprising virtually nothing more than potatoes and dairy products, poor oral health affected the quality of life for the poor of nineteenth‐century Ireland. This study investigates potential biocultural reasons that may explain why this was the case.

**Material and Methods:**

A total of 6,860 teeth and 9,889 alveoli from 363 permanent dentitions from the skeletal remains of impoverished adult Irish males and females who died between 1847 and 1851 in the Kilkenny Union Workhouse were examined for evidence of dental caries, periodontal disease and ante‐mortem tooth loss. Caries rates were quantified and assessed by crude prevalence, frequencies, corrected caries rates and a t‐health index, and evaluated by sex and age groups.

**Results:**

A higher rate of caries was present among 18–25‐year‐old males than females, while the opposite relationship was evident for older age groups. The prevalence rates of periodontal disease and ante‐mortem tooth loss increased with age. When assessed by corrected caries rates, tooth decay is observed at a lower rate compared to contemporaneous lower to upper‐class population samples from London.

**Discussion:**

Despite being low cariogenic foods, the potato starch and milk lactose of a nineteenth‐century Irish laborer's diet would have lowered oral pH‐values thereby increasing the risk of bacterial fermentation in dental plaque resulting in caries. Nutritional features alone cannot explain the high rates of dental caries observed in the Kilkenny workhouse population sample, however, and lifestyle factors, particularly habitual clay‐pipe smoking, is considered a significant cause of poor oral health.

## 1 INTRODUCTION

The laboring classes of nineteenth‐century Ireland subsisted on a monoculture economy based on potato agronomy (Feehan, 2012). At the beginning of the century the tuber had become the mainstay of the diet only occasionally, and depending on the season, supplemented with scanty additions of salt, pepper, and herring (Sexton, 2012). The potato was an essential crop for the poor or, as one contemporary commentator remarked, the “potato‐crop […] is in Ireland an affair of life or death. When it fails, a complete famine ensues; corn is out of the question, as the rich alone have the means of buying it” (Anon., 1842, p. 123). Potatoes thrived in the wet damp Irish climate and the crop was generally very productive. It has been estimated that the average laboring man in rural Ireland had a daily consumption of approximately 12 lbs (5.4 kg) of potatoes, while the quantities eaten by women and children were 10 lbs (4.5 kg) and 4 lbs (1.8 kg) or less, respectively. Potatoes were also used for making *poitín* (a traditional Irish distilled beverage) and were an important crop for feeding livestock (Bourke, 1968). While monotonous and repetitive, the potato diet—when supplemented by milk—was relatively nutritious providing sufficient calories and nutrients for the maintenance of health (Crawford, 1988). The reliance on a single crop for subsistence, however, led to catastrophic consequences during the latter half of the 1840s and early 1850s when blight *(Phytophthora infestans)* destroyed the only source of food for some 40% of the population (Kennedy, Ell, Crawford, & Clarkson, 1999). This period (1845–1852), generally referred to as the Great Famine or the Great Hunger, resulted in the death of nearly 1 million people; predominately the poor and the destitute (Ó Gráda, 2012).

While the overall nutritional value of the potato diet for the average agricultural worker in pre‐Famine Ireland is believed to have been relatively beneficial, there are also published accounts from the mid‐eighteen hundreds that describe Irish laborers rendered unfit for hard physical work due to their meager diet (see Boyle, 1983; Doyle, 1855). Food historian Margaret Crawford (1988) estimated the daily caloric intake for an adult male laborer to have been about 4,700 kcal. Most of these calories derived from carbohydrates in the potato, however, which is a quick‐digesting and fast‐burning source of energy (Kaur & Singh, 2016). According to Crawford (1988), the average diet only included an estimated daily 3.6 g of fat (deriving from milk). The dependence of high GI carbohydrates as an energy source explains why such large quantities of potatoes were consumed per capita in nineteenth‐century Ireland. The impact of the potato‐diet on oral health has not previously been considered. As the diet appears highly constant it may be hypothesized that the oral health of much of the working‐class Irish populace at the time would have been rather uniform. Current clinical knowledge would tend to suggest that the oral health of these people would have been generally good, as both the potato and milk are considered among the least cariogenic food products (Bowen & Lawrence, 2005; Moynihan, 2002; Moynihan & Petersen, 2007).

Contemporaneous descriptions of Irish oral health are contradictory and largely focused on culture and ethnicity as predisposing factors. For instance, Dr Frank Fuller (1827–1915)—a dental practitioner from Portsmouth, New Hampshire—stated that the Irish [in United States of America] “had, generally, poor teeth” due to the fact that “their diet was chiefly vegetable, and did not contain enough lime” (quoted in Anon., 1858, p. 82). Conversely, Dr John B. Rich (1811–1910), a practicing dentist from New York City in the late 1860s, was of the opinion that the Irish displayed “fine robust forms and strong teeth” and attributed this to “their simple food and out‐door work” (Horne, 1867, p. 197). Explanations advanced to account for variance in oral health patterns in the nineteenth century did not always focus on obvious factors relating to diet and lifestyle (although it has to be acknowledged that the general understanding of the causes of poor oral health at the time was limited, see Drummond, Wilbraham, & Hollingsworth, 1957, pp. 385–387). In many instances, they were based on racist stereotypes which, for the Irish, related to both ethnicity and religion (Finnegan, 2014). For instance, in 1885 Dr. S. Parsons Shaw (1825–1897), an eminent American‐born dental surgeon with a practice in Manchester, England (Anon., 1897), stated that the Irish “peasantry” had “coarse, large, and not good teeth,” descendants of English and Norse settlers in Ireland had “good and strong teeth,” while people of Scottish descent (i.e., Protestant) in the north of Ireland had “good, strong, and vital teeth” (Shaw, 1885, p. 79).

### 1.1 Aims and objectives

**Figure 1:**
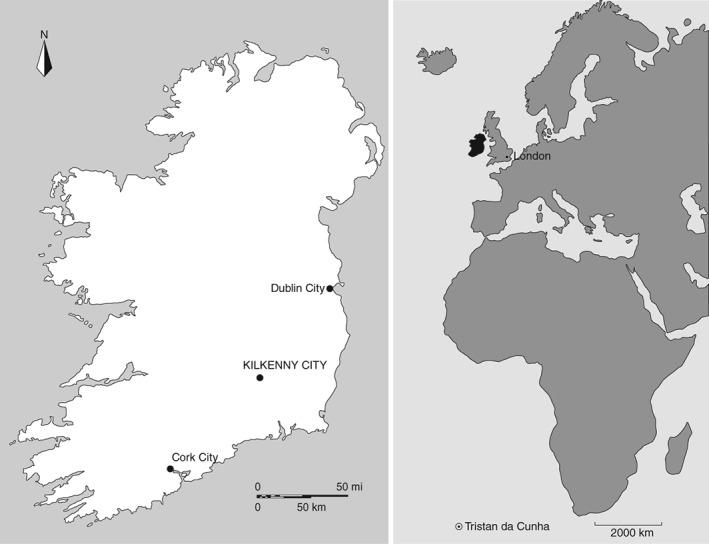
Location map of Ireland and the city of Kilkenny (left), and the geographical location of Tristan da Cunha (circled) in the South Atlantic (right)

This study investigates the oral health of a large sample of dentitions from adult males and females of the lowest social strata of mid‐nineteenth‐century Irish society. These people died and were subsequently interred in mass burials at the union workhouse in Kilkenny City in the south‐east of the island (Figure 1) at the height of the notorious Great Irish Famine (1845–1852). The aims and objectives of the study are to evaluate rates of tooth decay, periodontal disease, and ante‐mortem tooth loss to investigate whether the potato‐dominated diet of the Irish working class at this time generated a uniform or variable pattern of oral health. Other biocultural factors that may have contributed to dental disease in this population sample will also be explored. Unlike contemporary accounts, that are so clearly biased in multiple ways (see above), the study involves an objective examination of skeletal dentitions to address these issues. The discovery, and consequential study, of the Kilkenny Union Workhouse mass burials have generated a wealth of information relating to the experience of poverty, famine and social marginalization in Irish Victorian society (Geber, 2015). The assessment of oral health as a measure of the quality of life of the poor and destitute is an essential element to consider within these narratives (cf., Nikias, Fink, & Shapiro, 1975; Peres, Peres, de Barros, & Victora, 2007).

## 2 MATERIAL AND METHODS

The sample comprises a total of 6,860 permanent teeth and 9,889 alveoli derived from 363 dentitions. Only dentitions from adult skeletons that could be assigned a sex and specified age group were included in the study; these represented 192 males and 171 females. The individuals died as inmates of the Kilkenny Union Workhouse between August 1847 and March 1851, and their bodies were interred in a mass burial ground discovered in 2005 (Ó Drisceoil, 2005) and excavated in 2006 (O'Meara, 2006). In total, the Famine‐period mass burial ground at the Kilkenny Union Workhouse contained the remains of a minimum of 970 individuals (545 nonadults and 425 adults) (Geber, 2015). Isotope analysis of a subsample (*n =* 13) have revealed the population group subsisted on a more or less exclusively vegetarian diet (Beaumont et al., 2013; Beaumont & Montgomery, 2016), which conforms to nineteenth‐century historical accounts of the diet of the poor in this part of Ireland (Tighe, 1802). Preliminary analysis of dental calculus (Geber, 2016) has also revealed that, although potato and dairy products were largely consumed, the diet was supplemented with other vegetables (probably opportunistically and possibly related to relief food rations during the Famine period).

The bones and teeth were generally well preserved but several skeletons had been damaged by postburial factors including coffin collapse, soil compression, water erosion, bioturbation, and truncation. These factors reduced the dental sample to 85% of all adults of known sex. The bone preservation of skeletons with dentitions ranged from heavy surface erosion (Grade 5 in McKinley, 2004) to excellent condition (Grade 0 in McKinley, 2004), with the majority (*n =* 193; 53.2%) having a good to very good (Grades 2 and 1 in McKinley, 2004) state of preservation. No statistically significant difference in bone preservation was evident between male and female skeletons and dentitions (χ^2^(5) = 2.906, *p* = .751). A difference was noted across the age groups (χ^2^(15) = 57.395, *p* < .001), however, with a gradually declining state of bone preservation correlating with older age‐at‐deaths; this fact is unlikely to have significantly influenced the quantitative analysis of the current study.

### 2.1 Osteological analysis and diagnosis of dental disease

The skeletons were analyzed following standard osteological protocols. Age‐at‐death was estimated on the basis of the morphological characteristics of the pubic symphyses and auricular surfaces of the coxal bones (Brooks & Suchey, 1990; Lovejoy, Meindl, Pryzbeck, & Mensforth, 1985), the sternal ends of the ribs (İşcan & Loth, 1986; İşcan, Loth, & Wright, 1984), as well as late‐stage epiphyseal fusion in young adults (Scheuer & Black, 2000), and stages of cranial suture obliteration in older adults (Meindl & Lovejoy, 1985). Each adult skeleton was assigned to one of four age categories: 18–25 years (young adult), 26–35 years (early middle adult); 36–45 years (late middle adult) or ≥ 46 years (older adult) in accordance with the Museum of London Human Osteology Method Statement protocol (Powers, 2012) and agreed standard praxis in Irish osteoarchaeological research (Irish Association of Professional Osteoarchaeologists [IAPO], 2007). Sex was determined on the basis of cranial and pelvic morphological features (Buikstra & Ubelaker, 1994; Sjøvold, 1988). The teeth and alveoli were assessed and quantified using the two‐digit system devised by the Fédération Dentaire Internationale (FDI) (1971).

Dental caries was identified macroscopically with the occasional aid of a dental probe, and diagnosed and classified by relative size (Grade 0 = no lesion; Grade 1 = slight destruction of enamel/neck/root surface; Grade 2 = destruction of enamel/neck/root surface with pulp exposure; Grade 3 = gross destruction of enamel/neck/root surface with pulp exposure), type (pit and fissure; smooth surface; root) and surface location for each tooth (Hillson, 2001; Lukacs, 1989; Scheid & Weiss, 2012, pp. 291–294). Ante‐mortem tooth loss was recorded as either present or absent for each alveolus and diagnosed based on the presence or absence of substantial bone remodeling or complete obliteration of a socket. Slight, moderate to considerable extents of dental calculus (mineralized plaque) and periodontal disease (Ogden, 2008; Schultz, 1988), as well as the general extent of attritional wear (Brothwell, 1981), were assessed for each dentition on an overall level and not by individual teeth or sockets. In addition, peri‐apical abscesses (classified in accordance with Dias & Tayles, 1997) and pipe‐smoker's notches were also recorded. Frequency data for caries (any size and surface location) and ante‐mortem tooth loss were calculated for each observable alveolus and tooth, and crude prevalence rates per individual were determined, when at least one quarter of a dentition was present.

### 2.2 Caries correction rate (CCR)

The rate of tooth decay was also calculated using the Caries Correction Factor method devised by Lukacs (1995) to facilitate more authentic results. This method considers the likelihood that cases of tooth loss are a consequence of a decayed tooth. Dental frequency rates are adjusted by a factor calculated from the proportion of observable teeth that display pulp exposure due to tooth decay rather than noncarious factors, such as dental attrition. No cases of pulp exposure due to dental attrition were observed in the sample which is reflective of the soft potato diet. The lack of abrasive dietary components is further evidenced by a low frequency of enamel chipping (see Scott & Winn, 2011; Turner & Cadien, 1969) in the permanent teeth. This was only observed in 0.4% of incisors (*n =* 6/1,606), 0.1% of premolars (*n =* 1/1,977) and 0.3% of molar teeth (*n =* 7/2,270), although the true rate of dental trauma is likely to be significantly obscured due to the occurrence of gross carious lesions. As a result, the calculation of the corrected caries rate (CCR) in this population sample becomes identical to the Diseased Missing Index (DMI) occasionally used in dental anthropological research (e.g., Prowse, 2011).

A limitation of the Caries Correction Factor method is its presumption that dental caries or considerable attrition is the prime cause of ante‐mortem tooth loss. Clinical research clearly demonstrates dental caries to be one of the main etiologies of tooth loss, accounting for approximately 50% of cases (including intentional extractions), but periodontal disease has been identified as the main cause of tooth loss in 30%–35% of cases (Papapanou, 1996). A binary logistic regression analysis by dentitions confirmed that both caries and periodontal disease co‐occurred with ante‐mortem tooth loss at similar odds ratios (Table 1), but it is not possible to determine which condition would have been the main cause. Vitamin C deficiency (scurvy) (Brickley & Ives, 2008, pp. 48–49) is a possible noncarious‐related cause of tooth loss of relevance for this population sample. This condition has previously been diagnosed in high rates in these individuals (Geber & Murphy, 2012). A chi‐square test, however, failed to detect a difference in the frequency of tooth loss between nonscorbutic and scorbutic (possible, probable, and definite diagnoses) individuals for both anterior (χ^2^(1) = 0.140, *p* = .708) and posterior (χ^2^(1) = 0.005, *p* = .943) dentitions. As such, scurvy is an unlikely cause of tooth loss in this population sample.

**Table 1:** Binary logistic regression model for dental caries and periodontal disease as predictive variables for ante‐mortem tooth loss in the permanent dentitions of adult males and females

	B	S.E.	Wald	d*f*	Sig.	Exp(B)	95% CI Exp(B)
Dental caries	1.017	0.268	14.410	1	<.001	2.764	1.635–4.671
Periodontal disease	1.245	0.243	26.188	1	<.001	3.473	2.156–5.594
Constant	−3.179	0.550	33.358	1	<.001	0.042	

*R* ^2^ = 0.199 (Nagelkerke), 0.146 (Cox and Snell). Model χ^2^(2) = 55.355, *p* < .001.

### 2.3 T‐health index

As an additional measure of oral health, a t‐health index was calculated based on the weighed factors suggested by Bernabé, Souominen‐Taipale, Vehkalahti, Nordblad, and Sheiham (2009). This study evaluated 36 combinations of weights and compared them with the perceived oral health status of a Finnish clinical patient sample (*n =* 5,057) comprising individuals aged more than 30 years. An ordinal logistic regression analysis, that considered gender, age, education, income, diet, smoking, and oral hygiene habits, determined the best weight factor combination (T‐health‐10) in association with perceived oral health from the highest odds ratio value (OR = 1.60) obtained from the analysis. Barnabé and colleagues also concluded that the DMF‐index, commonly used in odontology (Klein & Palmer, 1938), displayed the lowest odds ratio (OR = 0.66), and was therefore, the least appropriate measure of oral health perception in the patient sample. As the t‐health index can only be calculated from dentitions with no postmortem tooth loss, the method has major limitations when applied to archaeological samples where postdepositional loss of teeth is a standard occurrence. To increase the dataset for the study, and to exclude agenesis as a potential cause of missing teeth (see Hillson, 1996, pp. 113–114), the t‐health index calculation excluded third molars. As such, some 28 teeth/alveoli were assessed for each dentition which generated t‐health‐index values ranging from 0.00 (all teeth lost) to 28.00 (all teeth present and noncarious). In addition, separate t‐health indices were calculated for the anterior and posterior dentitions.

### 2.4 Statistical analysis

All statistical analyses were conducted using the SPSS statistical software package (IBM Corp. Released 2016. IBM SPSS Statistics for Mac, Version 24.0). Chi‐square tests were undertaken to determine significant differences in rates and frequencies between sexes and age groups, and binary logistic regression analysis was conducted to observe correlation patterns. A Kruskal‐Wallis H‐test was used to investigate age‐related difference in crude prevalence rates across age groups. Nonmetric correlation analyses were undertaken using the Spearman Rho‐test. Detailed frequencies of teeth affected by caries and ante‐mortem tooth loss are available in Supporting Information Tables S1 and S2.

## 3 RESULTS

**Figure 2:**
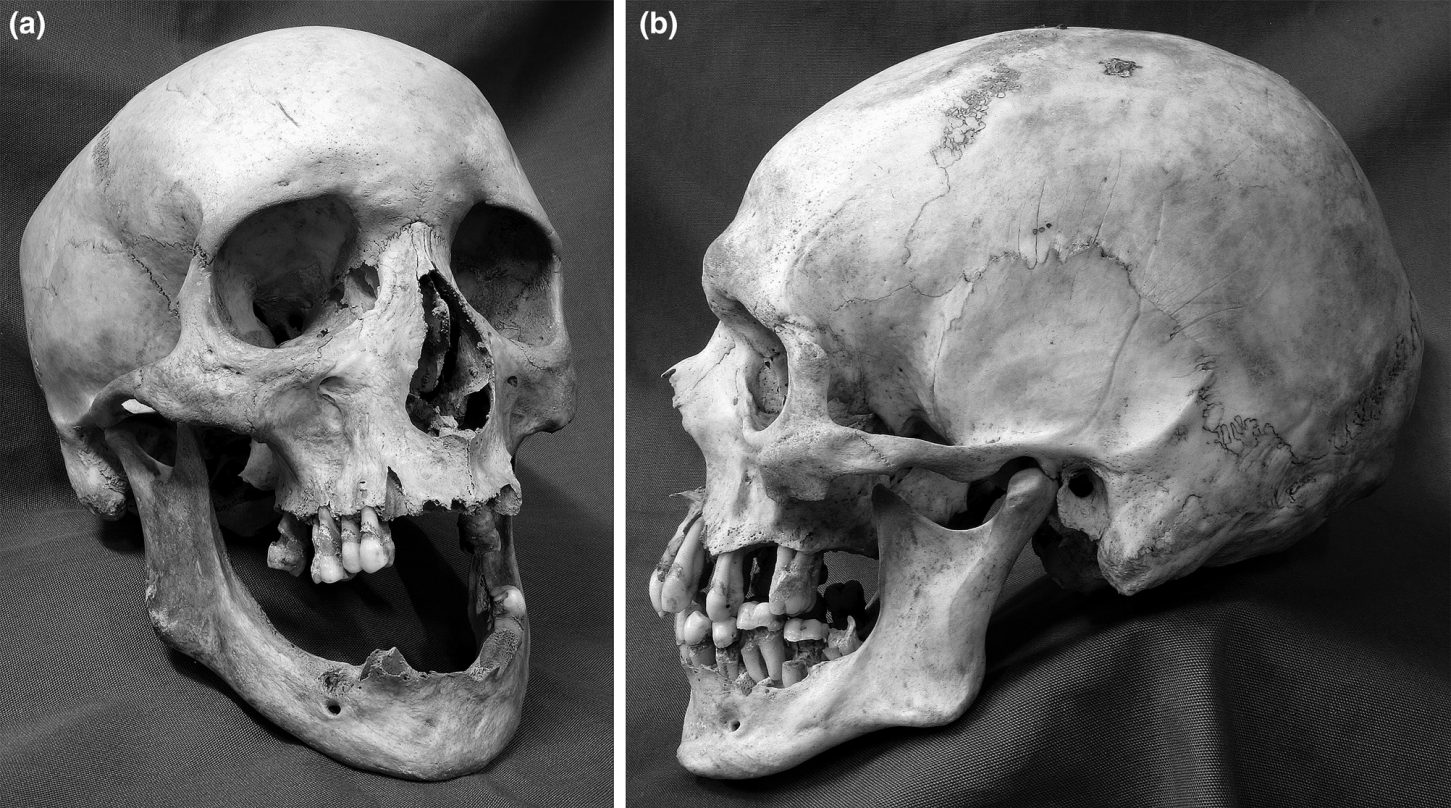
Examples of dental pathological conditions in adult crania from the Kilkenny Union Workhouse mass burials: (a) 26–35‐year‐old female (DCCCII) with evidence of ante‐mortem tooth loss, dental caries, dental calculus and periodontal disease; (b) 36–45‐year‐old male (CCLXXXII) with evidence of dental caries, dental calculus, and severe periodontal disease. Photo: Jonny Geber

The crude prevalence rates of dental conditions (Figure 2) in the Kilkenny Union Workhouse adults are summarized in Table 2. Nearly 80% of adult dentitions exhibited evidence of tooth decay, while periodontal disease and periapical abscesses were present in 58% and 27% of adult dentitions, respectively. Tooth loss was observed in over half, while calculus was visible in over 96% of adult dentitions.

**Table 2:** Crude prevalence (%) of dental conditions in adult male and female dentitions

Sex	Tooth lesions				Jaw lesions		
	Caries	Enamel hypoplasia	Calculus	Clay‐pipe facets	AMTL^1^	Periodontitis	Periapical lesions
Males	83.7	19.2	97.7	60.7	60.3	62.5	28.6
	[*n =* 144/172]	[*n =* 35/182]	[*n =* 171/175]	[*n =* 99/163]	[*n =* 114/189]	[*n =* 120/192]	[*n =* 55/192]
Females	75.5	24.6	96.9	28.6	68.3	52.0	24.7
	[*n =* 114/151]	[*n =* 41/167]	[*n =* 154/159]	[*n =* 40/140]	[*n =* 114/167]	[*n =* 89/171]	[*n =* 72/170]
Total	79.9	21.8	97.3	45.9	64.0	57.6	26.8
	[*n =* 258/323]	[*n =* 76/349]	[*n =* 325/334]	[*n =* 139/303]	[*n =* 228/356]	[*n =* 209/363]	[*n =* 97/362]

1 Ante‐mortem tooth loss.

### 3.1 Prevalence rates of dental caries

**Figure 3:**
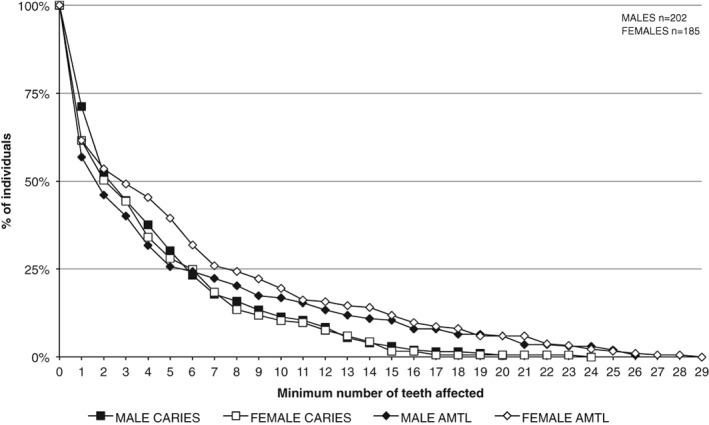
The cumulative proportion of adult males and females according to the minimum number of teeth affected by dental caries and tooth loss in each dentition

The rates of the total number of permanent teeth affected by caries ranged from 4% (*n =* 34/914) in 18–25‐year‐olds to 29% (*n =* 255/873) in those aged ≥46 years (although not investigated as part of this particular study, it is worth mentioning the rate of teeth affected by caries in the nonadult age groups of this population sample: 1–5 years [0.9%, *n =* 26/2,862]; 6–12 years [2.2%, *n =* 59/2,695]; 13–17 years [1.4%, *n =* 17/1,174]). A statistically significant difference between male (M) and female (F) frequency rates was observed for the 18–25 years age group (M > F), and the 26–35 years and ≥ 46 years age groups (M < F). These unadjusted prevalences are likely to be a misrepresentation of the true situation; however, as ante‐mortem tooth loss rates by number of alveoli were substantially and significantly higher among females across all age groups (Table 3). This trend is further indicated from an assessment of the minimum number of teeth affected by caries or ante‐mortem tooth loss between male and female dentitions (Figure 3). While no significant difference was evident between male and female dentitions in relation to the average number of teeth affected by caries (χ^2^(20) = 22.757, *p* = .301), a significantly higher proportion of females had lost more teeth per dentition than males (χ^2^(28) = 49.475, *p* = .007). The greatest difference between the sexes was observed in dentitions with less than 10 teeth affected by tooth loss, after which they exhibited similar rates.

**Table 3:** Frequency of total number of permanent teeth affected by dental caries, and the total number of alveoli for permanent teeth indicating ante‐mortem tooth loss, by age group (years) and sex

Age group	Carious teeth						Teeth lost ante‐mortem					
	Males		Females		χ^2^ (d*f* = 1)	*p* value	Males		Females		χ^2^ (d*f* = 1)	*p* value
	%	No./Total	%	No./Total			%	No./Total	%	No./Total		
18–25	6.3	31/493	0.7	3/421	19.709	<.001	0.6	3/526	3.8	18/470	12.776	<.001
26–35	12.0	115/962	15.4	211/1,370	5.585	.018	3.9	46/1,168	11.9	210/1,759	56.289	<.001
36–45	25.3	386/1,527	27.5	334/1,214	1.743	.187	20.2	481/2,378	25.3	497/1,965	15.823	<.001
≥46	27.4	189/691	36.3	66/182	5.533	.019	27.3	319/1,167	46.7	213/456	55.863	<.001
Total:	19.6	721/3,673	19.3	614/3,187	0.144	.704	16.2	849/5,239	20.2	938/4,650	26.181	<.001

**Figure 4:**
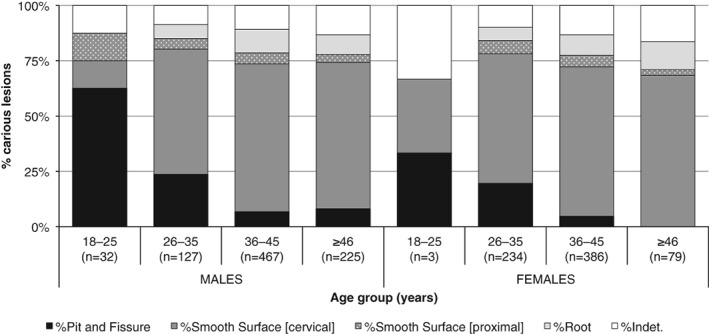
The relative proportion of pit and fissure, smooth surface, and root caries in the total number of carious lesions in adult males and females, by age groups

When assessed by type, the proportions of pit and fissure caries were highest in the 18–25 years age group for both sexes (Figure 4), and the general trend suggests a decrease with advancing age. The opposite relationship was observed for root caries which increased with age. These findings are consistent with an expected trend, relating to low rates of periodontal disease and root exposure (resulting in root caries) among young adults, and a higher rate of molar tooth loss (resulting in fewer pit and fissure sites) and periodontal disease (which increase the rates of root exposure) among older adults (Darby & Walsh, 2010, pp. 243–245; Mobley, 2007). The most common sites for caries were smooth tooth surfaces, particularly at the neck (cervical caries). The proportion of smooth surface caries increased beyond 25–35 years for both males and females, after which it remained relatively constant. Overall, the relative distribution of the sites of carious lesions was similar between males and females. The only exception was for the 18–25 years age group in which a very low rate of caries was present in female dentitions, while the distribution in males showed a low proportion of smooth surface caries.

#### 3.1.1 Corrected caries rates and t‐health index values

When assessing the data using the Corrected Caries Rate, which takes ante‐mortem tooth loss into account, a significantly higher rate of caries in adult female teeth was evident across the age groups, with the exception of the 18–25 years age category in which no statistically significant difference was observed (Table 4). The biggest disparity between the sexes was observed for the 26–35 years age group, while the least substantial difference arose in the subsequent age category. To determine whether any specific pattern of caries had affected teeth in male and female dentitions the corrected caries rate was calculated separately for anterior (incisors and canines) and posterior (premolars and molars) teeth. The results indicated a substantial difference between male and female dentitions in relation to the increase in caries rates in the anterior dentition between the 26–35 years and 36–45 years age groups. The rate increased by 2.3 times in females, while in male dentitions it was substantially higher at 5.7. No such difference was observed in the posterior dentitions.

**Table 4:** Corrected caries rates calculated separately for anterior and posterior permanent teeth in adult male and female dentitions, by age group (years)

Age group	Anterior teeth				Posterior teeth				All teeth			
	Males	Females	χ^2^ (d*f* = 1)	*p* value	Males	Females	χ^2^ (d*f* = 1)	*p* value	Males	Females	χ^2^ (d*f* = 1)	*p* value
18–25	2.3	0.0	3.726	.054	9.4	6.9	1.247	.264	6.9	4.8	1.805	.179
26–35	6.0	16.3	21.028	<.001	21.3	32.4	214.033	<.001	16.0	26.6	40.219	<.001
36–45	34.4	34.9	0.041	.839	48.1	56.0	15.061	<.001	43.2	48.6	10.820	.001
≥46	41.4	60.4	150.48	<.001	55.3	76.5	34.046	<.001	50.3	70.6	47.660	<.001
Total:	26.3	26.6	0.029	.866	39.4	43.8	11.140	.001	34.7	37.6	7.892	.005

It was possible to calculate the t‐health index from 60 individuals and it ranged from 0.2 in a 25–35‐year‐old female (DCCXCVI)—who was completely edentulous with the exception of one remaining carious tooth in the upper dentition—to 28.0 in eight male and 16 female dentitions. Although a calculation of the t‐health index requires complete dentitions, and the analytical data for the study was therefore significantly reduced, a comparison of indices between males and females across the age groups is interesting. The available data does not reveal any significant difference in oral health status between the sexes (Table 5), even though the corrected caries rates were highly indicative of such a trend.

**Table 5:** T‐health‐index (10) values, by sex and age group (years)

Age group	Males					Females					χ^2^	d*f*	*p* value
	$\bar{x}$	Min.	Max.	SD	*N*	$\bar{x}$	Min.	Max.	SD	*N*			
18–25	26.9	24.6	28.0	1.4	7	27.2	22.2	28.0	2.2	7	6.000	4	.168
26–35	25.8	19.9	28.0	2.6	9	23.7	0.2	28.0	7.2	16	9.375	11	.863
36–45	20.2	1.0	28.0	9.0	10	24.5	14.9	28.0	4.7	7	12.046	11	.508
≥46	19.9	8.1	25.3	8.0	4	–	–	–	–	0	–	–	
Total	23.4	1.0	28.0	6.6	30	24.7	0.2	28.0	5.9	30	26.667	25	.373

### 3.2 Calculus, periodontal disease, and tooth loss

Calculus was present at consistently high rates in dentitions of all age groups (18–25 years: 97.0%, *n =* 32/33; 26–35 years: 97.1%, *n =* 99/102); 36–45 years: 97.9%, *n =* 141/144; ≥46 years: 96.4%, *n =* 53/55). The prevalence of more severe calculus deposits increased with age, however, in both male (χ^2^(3) = 8.885, *p* = .031) and female (χ^2^(3) = 17.809, *p* < .001) dentitions. The high rate of calculus is primarily an indicator of poor oral hygiene as these are only apparent if dental plaque is not removed from the teeth on a regular basis (Greene & Vermillion, 1960). The etiology of calculus is multifactorial, however, and dietary factors, such as food stuffs high in carbohydrates and/or proteins that elevate pH‐values in the oral environment, would also influence its formation (Roberts & Manchester, 2005, pp. 71–73). Furthermore, an individual's salivary flow rate may relate to the dietary intake of fluids through increased hydration and might potentially elevate mineral levels in the oral cavity (Lieverse, 1999). No significant difference in the crude prevalence of calculus deposits was observed between male and female dentitions (χ^2^(1) = 0.234, *p* = .628), and it is therefore likely that men and women in this population sample had similar oral hygiene practices.

**Figure 5:**
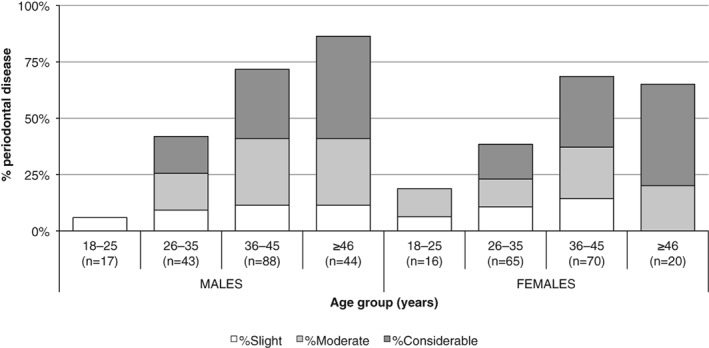
The crude prevalence rate of periodontal disease by severity in adult males and females, by age groups

Crude prevalence rates of periodontal disease increased with age, and it was observed in 12% (*n =* 4/33) of dentitions within the 18–25 years age category, 40% (*n =* 43/108) in the 26–35 years age category, 70% (*n =* 111/158) in the 36–45 years age group, and 80% (*n =* 51/64) in the ≥46 years age group (χ^2^(3) = 64.889, *p* < .001). The proportion of slight manifestations of periodontal disease had a relatively even distribution across the age groups for both sexes, while the occurrence of considerable levels of the condition increased with age (Figure 5). With the exception of the 18–25 years age category, males displayed a higher rate of periodontal disease than females. Among females, the rate of periodontitis was slightly less in the ≥46 years age (65.0%; *n =* 13/20) group than in the preceding age category (68.6%; *n =* 48/70), although this difference was not statistically significant (χ^2^(1) = 0.091, *p* = .763). A correlation between increased severity of calculus deposits and advanced periodontal disease was only observed among males aged 25–36 years (*r*_s_ = .313, *p* = .044). This would suggest that, while calculus very likely affected the rate of periodontal disease in this population sample (Williams, 1990), it was probably not the main causative factor.

Ante‐mortem tooth loss (≥1 tooth lost) was observed at crude prevalence rates in 18% (*n =* 6/33) of 18–25‐year‐olds, 51% (*n =* 53/104) in 26–35‐year‐olds, 74% (*n =* 115/156) in 36–45‐year‐olds, and 86% (*n =* 55/63) in those aged ≥46 years. Females were more affected than males (see Table 2), although this difference was not statistically significant (χ^2^(1) = 2.431, *p* = .119). Molars were the teeth most affected in both males and females (Supporting Information Table S2), and the pattern broadly follows the trend for the teeth most affected by carious lesions (Supporting Information Table S1). This suggests there is a relationship between tooth decay and tooth loss in this population sample.

## 4 DISCUSSION

Tooth decay or the formation of dental caries is a multifactorial process, that relates to underlying oral microbiology, salivary flow and composition, tooth morphology, the retention time of food in the oral cavity, eating habits, oral hygiene, fluoride exposure, as well as social factors such as family structure and socioeconomic background (Darby & Walsh, 2010; Frazão, 2012; Selwitz, Ismail, & Pitts, 2007; Touger‐Decker & van Loveren, 2003). It is a chronic destructive condition that generally progresses slowly and is caused by demineralization of the enamel, dentine, and cement of the teeth due to the acid bacterial production *(Streptococcus mutans et sobrinus)* that occurs during fermentation of carbohydrates in plaque bacteria (Hillson, 1996, 2005; Mobley, 2007; Selwitz et al., 2007). In addition to causing pain and discomfort, tooth decay can expose a tooth to further infection, and several clinical studies have reported a positive correlation with an increased mortality risk in individuals with poor oral health (Jansson, Lavstedt, & Frithiof, 2002; Kim, Baker, Davarian, & Crimmings, 2013). A significant relationship has also been demonstrated between poor oral health and low socioeconomic status in numerous modern studies across the world (Chandra Shekar & Reddy, 2011; Dye & Thornton‐Evans, 2010; Hobdell et al., 2003; Park, Han, Park, & Ko, 2016; Paula et al., 2012). It is clear from the current study that the poor in mid‐nineteenth‐century Ireland also suffered from oral health problems.

Depending on the method of quantification, the male to female ratio of caries/oral health rates, and its statistical significance value, differ noticeably. Overall, the general trend suggests that oral health was worse for males than females in the 18–25 years age category, but the opposite relationship was true for the ≥26 years age groups. This observation is interesting as it differs from most clinical and bioarchaeological assessments of oral health patterns which generally indicate a consistently worse female oral health status across all adult age groups (Hollander & Dunning, 1939; Lukacs, 2011; Wasterlain, Hillson, & Cunha, 2009). A higher caries frequency in female dentitions is consistent with the well‐known biological bias, relating to hormonal differences during puberty, pregnancy, and menopause in particular. Hormonal fluctuations during these periods have negative effects on the composition and flow of saliva which creates an increased risk of dental caries (Lukacs & Largaespada, 2006).

### 4.1 Cariogenicity of the potato and milk diet

Diet is unlikely to have been a dominant cause of the high rates of tooth decay in this population sample. In relative terms, potatoes are a low cariogenic food group generally considered not harmful to teeth (Bibby, 1961; Moynihan, 2002), even though a large proportion of the tuber comprises carbohydrates. Potato carbohydrates can be classified into three components: starch, which constitutes approximately 60%–80% of the potato in dry weight (DW); nonstarch polysaccharides that form only about 6% of DW; and sugars (sucrose, fructose, and glucose), which are only present in very small amounts (~ ≤3.0% of DW) depending on the variety of potato and storage conditions (Duarte‐Delgado et al., 2016; Schwimmer, Bevenue, Weston, & Potter, 1954; Woolfe, 1987, pp. 10–12). Boiled potatoes have a higher cariogenic risk factor, however, mainly because they contribute to a significant drop in oral pH that is retained at this low level for a relatively long time (Lingström, Birkhed, Granfeldt, & Björck, 1993; Lingström, van Houte, & Kashket, 2000). The low pH in combination with the general propensity of potato to cling onto the teeth (Bibby, 1961) could, in turn, increase the risk of dental demineralization (Hillson, 1996). Even after considering all of these factors and acknowledging that potatoes do have cariogenic potential; however, it is important to appreciate that they are still among the food groups that have the least impact on dental caries rates. Indeed, they have been advocated within dietary advice given to patients wishing to avoid tooth decay (Moynihan, 2002).

The principal carbohydrate in milk—the main liquid component of the Irish pre‐Famine diet—is lactose, which comprises ~ 5% of its weight (Scrimshaw & Murray, 1988). Lactose is the least cariogenic of the most common dietary sugars; however, and the beneficial concentrations of calcium and phosphorous in milk—which helps to protect dental enamel—has caused dental clinicians to advocate milk consumption as a preventive action against tooth decay (Johansson, 2002; Rugg‐Gunn & Petersen, 2009). In a similar manner to potato starch, milk significantly reduces oral pH values (Birkhed, Imfeld, & Edwardsson, 1993; Johansson, 2002), and may therefore potentially (although minorly so) contribute to an increased risk of bacterial fermentation in dental plaque. A soft diet based on potatoes and milk products would also have required less chewing, and thereby a decreased salivary flow rate and oral clearance, which may be another influencing factor in caries formation (Mobley, 2007). Overall, nutritional factors alone, however, would not explain the high rates of dental caries observed in the Kilkenny workhouse population sample.

In early 1926, during one of the expeditions of the Antarctic research vessel *R.R.S. Discovery* (Savours, 2001), Dr. Edward Hillis Marshall (1885–1975), the ship's surgeon, undertook a dental examination of the population living on the remote island of Tristan da Cunha in the south Atlantic (see Figure 1). Dr. Marshall examined a third (*n =* 54) of the entire population (then comprising 141 individuals) and observed remarkably low caries rates and an excellent state of oral health. Caries frequencies by number of teeth ranged from 0% in 15–20‐year‐olds to only 6% in 45–99‐year‐olds, and the prevalence of carious mouths ranged from 0% to 67% for the same age groups. Marshall's study is an ideal comparison to the pre‐Famine Irish context as the diet of the populace on Tristan da Cunha at the time of his examination almost entirely comprised potatoes and milk. The only notable difference was the islanders' exploitation of seabirds and their eggs (mollymawks and penguins in particular). Seafood did not appear to be popular despite an abundance of easily caught fish (Marshall, 1926). Potato and milk still remained a large proportion of the diet of the Tristan da Cunha islanders in the late 1950s (Taylor, Hollingsworth, & Chambers, 1966), although from the time of Marshall's visit (1926) and onward, there was an increased consumption of sugar and grains on the island that concomitantly increased the prevalence and frequency of dental disease (Barnes, 1937; King‐Turner & Davies, 1956; Sampson, 1932).

**Figure 6:**
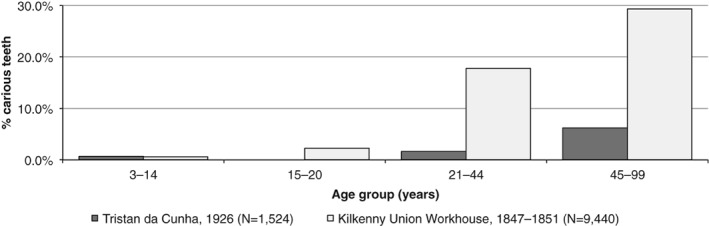
The frequency of teeth affected by dental caries in the residing population of Tristan da Cunha in January 1926, compared to the frequency observed in the Kilkenny Union Workhouse population sample (dating to between August 1847 and March 1851)

Dr. Marshall also commented upon the oral hygiene of the populace, and stated that “in no case did anyone admit to cleaning his or her teeth more often than once a week,” and that “it is probably correct to say that the islanders, as a whole, never clean their teeth” (Marshall, 1926, p. 1102). When quantified by carious tooth frequencies, the highest rates of caries in the Tristan da Cunha population were evident among 45–99‐year‐olds at 6%. This is exceptionally low (χ^2^(1) = 75.811, *p* < .001) compared to the Kilkenny Union Workhouse samples, where the frequencies for the same age category was 29% (Figure 6). The fact that these two populations, who subsisted on a virtually identical diet, display such significantly different oral health patterns is highly suggestive that other nondietary factors are the major cause of tooth decay in the Kilkenny workhouse population.

### 4.2 Susceptibility and lifestyle factors

**Figure 7:**
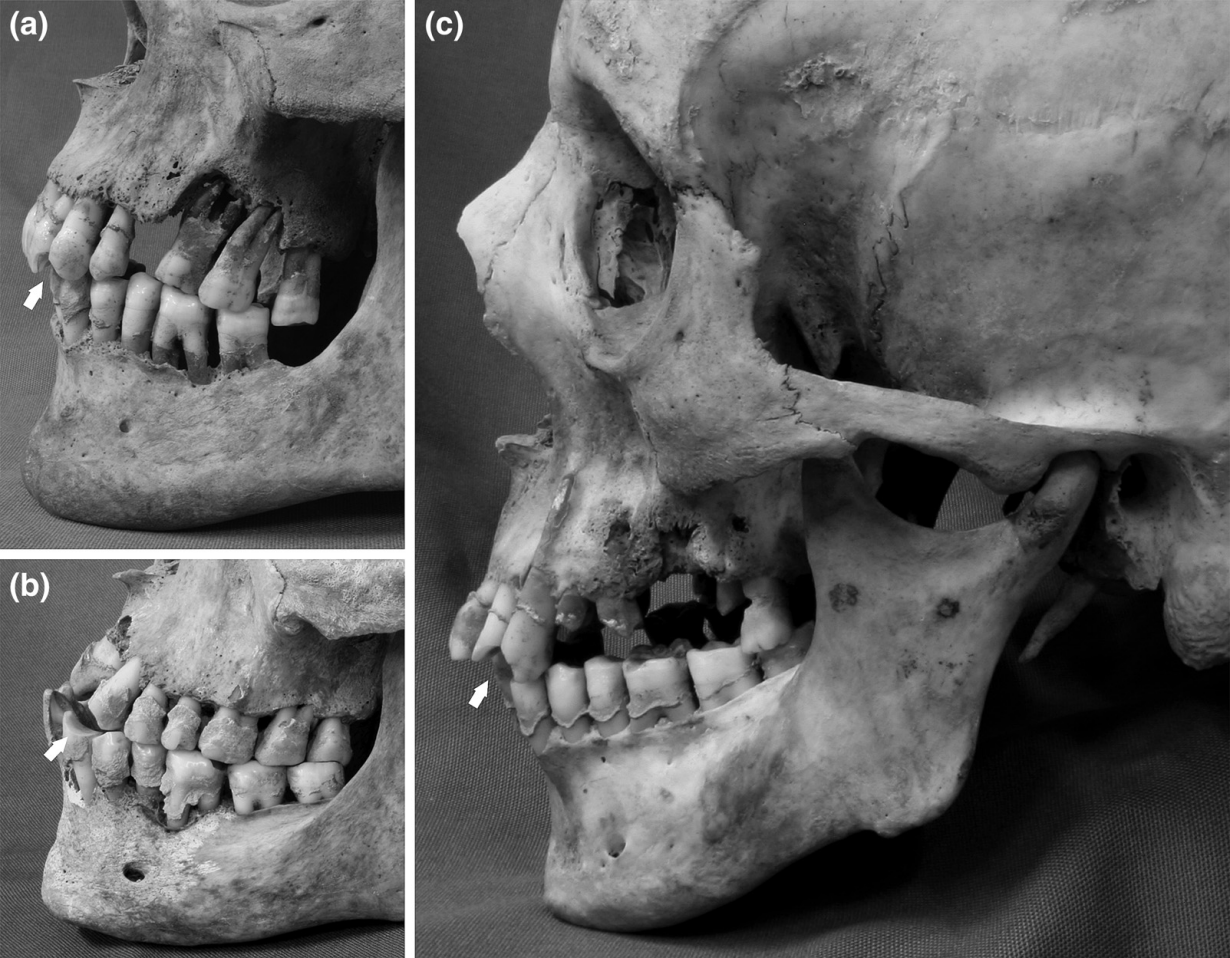
Examples of clay‐pipe facets (arrows) manifested in adult dentitions: (a) ≥46‐year‐old male (DCCLXV) with evidence of ante‐mortem tooth loss, dental caries, dental calculus and periodontal disease; (b) 36–45‐year‐old male (DCCLXXXV) with evidence of ante‐mortem tooth loss, dental calculus and ante‐mortem tooth loss; and (c) 26–35‐year old male (CCLXXXV) with evidence of ante‐mortem tooth loss, a periapical abscess, dental caries, dental calculus, and periodontal disease. Photo: Jonny Geber

It is evident, from the high frequency of clay‐pipe facets in the dentitions (Figure 7), that a large proportion of both adult males and females who died in the Kilkenny Union Workhouse were habitual smokers (Geber, 2015). This habit is likely to have had negative impacts on their oral health (Axelsson, Paulander, & Lindhe, 1998; Smejkalova et al., 2012). Danaher (1962, p. 67) makes reference to stories of travelers to Ireland in the nineteenth century who recounted with disapproval that all of the boys, and many of the girls, aged around 14 years were pipe smokers, while early nineteenth‐century economists concerned with resolving Ireland's problems complained that the “common laborers” spent up to a half‐penny a day on tobacco. On his visit to the city of Cork in 1796–1797, the French Royalist Cavalry Officer, Jacques‐Louis de Bougrenet de la Tocnaye (1767–1823), paints a poignant picture of the poor and their smoking habits: “Although the people are very poor, nothing or no one can persuade the mothers to send their children to the poorhouse or almshouse. […] A frequent site is one of these poor unfortunates with two children on her back and another in her apron, holding another by the hand, and beseeching for the cold charity of the passers‐by, who being accustomed to such sights generally turn away their eyes. The poor woman, however, also accustomed to such indifference, consoles herself by smoking a black pipe, so short that the fire almost seems to be in her mouth” (de Bougrenet de la Tocnaye, 1917, p. 37).

Smoking causes both thermal and chemical‐induced damage to periodontal tissues at a relatively rapid pace. It is also associated with immunodepressive effects due to decreased production of salivary antibodies and impaired neutrophil functioning, and an impaired buffering effect of the saliva which increases susceptibility to tooth decay (Darby & Walsh, 2010, p. 306; Voelker, Simmer‐Beck, Cole, Keeven, & Tira, 2013). Clinical studies have shown a positive correlation between smoking and periodontal disease (Bergstrom, 2014; Borojevic, 2012; Smejkalova et al., 2012) and caries rates (Aguilar‐Zinser et al., 2007; Voelker et al., 2013), although these links are also considered to be inconclusive (U.S. Department of Health and Human Services, 2014). Additional lifestyle factors that are often socioeconomic in nature, including different eating habits and oral hygiene maintenance, have also been shown to differ substantially between smokers (including secondhand smoking) and nonsmokers (Benedetti, Campus, Strohmenger, & Lingström, 2013; Hanioka, Ojima, Tanaka, & Yamamoto, 2011; Smejkalova et al., 2012). The pattern of tooth decay in smokers is influenced by the increased risk of periodontal disease and caries is more frequently observed at, and below, the gingival junction of the teeth (Bharateesh & Kokila, 2014; Rad, Kakoie, Brojeni, & Pourdamghan, 2010; Soetiarto, 1999).

Periodontal disease, cervical and root caries frequently affected the population sample from Kilkenny and it seems very plausible that these occurred as a consequence of habitual smoking. This can be argued from chi‐square tests, which indicate a significant link between the progression of periodontal disease (slight, moderate, severe) and the presence of clay‐pipe facets (χ^2^(3)= 8.903, *p* = .031), and caries at and below the gum line (cervical and root caries grouped together) (χ^2^(3)= 65.777, *p* < .001). Another suggestive relationship between smoking and oral health can also be discerned from the age‐specific pattern of the prevalence of clay‐pipe facets and the relative proportion of the different types of caries between males and females; for both sexes, the rates for both variables are relatively uniform for the ≥26 years age groups, while a discrepancy appears to be present in the 18–25‐year‐olds. The crude prevalence of clay‐pipe facets in male dentitions ranged from 24% (*n =* 4/17) in 18–25‐year‐olds to 80% (*n =* 28/35) in ≥46‐year‐olds, while the female rates ranged from 0% (*n =* 0/16) to 50% (*n =* 6/12) in the corresponding age groups. This may indicate that females started to smoke a pipe at an older age than males and the consumption pattern of tobacco had gender‐related norms within the population. It may also be the reason why a differential pattern in carious attacks is observed between young adult males and females, but not in the other age categories.

Another lifestyle factor that may have influenced caries rates is alcohol consumption. Although alcohol is not cariogenic as such (Dukić, Trivanović Dobrijević, Katunarić, & Lešić, 2013; Harris, Warnakulasuriya, Gelbier, Johnston, & Peters, 1997), it results in inhibited salivary flow and mouth dryness (Cox & Ship, 2008, p. 210), which in turn increases the risk of developing carious lesions, particularly cervical and root caries (Challacombe, Osailan, & Proctor, 2015). Returning to the comparable early twentieth‐century populace of Tristan da Cunha, a study by Norwegian sociologist Peter A. Munch (1908–1984) in the late 1930s, remarked upon the fact that the islanders did not drink alcohol (Munch, 1945, p. 68). Conversely, the brewery industry was one of the main components of the Irish economy in the nineteenth century (Lee, 1966; O'Malley, 1981) and alcohol was clearly consumed, although not to the extent implied in the racist stereotype of the “drunken Irish,” popularized in the British and American press at the time (de Nie, 2004; Ferriter, 2015; Hirsch, 1991; Stivers, 1976). The temperance movement in Ireland was very strong during the first half of the nineteenth century, largely due to the efforts of Father Theobald Mathew (1790–1856), a Catholic Priest and temperance reformer who personally, through his social work, persuaded hundreds of thousands of people across Ireland to give up drink for the rest of their lives (Kelly, 1994). In 1841, when Ireland had a population of about 8 million inhabitants, it was stated that the Irish temperance movement had over 5 million members, although this is likely to be an exaggeration (Kelly, 1994; Lysaght, 1983).

### 4.3 Poverty and oral health

The Great Famine in Ireland was a terrible health insult to a population already exposed to considerable health risks relating to poverty and dire living conditions. The primary causes of death during the Famine were infectious diseases, which were common in mid‐nineteenth‐century Ireland, but resulted in mass death in this malnourished and starved population. It is possible that such underlying health issues, related to socioeconomic factors, had also influenced the risk of developing tooth decay in the inmates from the Kilkenny Union Workhouse long before the Famine. While several clinical studies have reported a positive correlation between malnutrition and caries in primary teeth (Alvarez & Navia, 1989; Ismail, 1998; Johansson, Saellström, Rajan, & Parameswaran, 1992), there is currently a limited understanding as to whether the same relationship exists for the permanent dentition. A review article published in 2005 (Psoter, Reid, & Katz, 2005) only identified one longitudinal case study from Peru (Alvarez et al., 1993), where a link between early childhood protein‐energy malnutrition (PEM) and caries in the permanent dentition was observed. This particular study argued that the same factors observed in experimental rat studies, where malnourishment due to PEM and Vitamin A deficiency resulted in impaired amelogenesis and reduced salivary flow rates, caused an increase in caries frequencies (Harris & Navia, 1980; Johansson, Ericson, Bowen, & Cole, 1985; Navia, 1979).

Access to oral health care is a further social factor that requires consideration. The origins of dental surgery in Ireland can be traced back to the late eighteenth century, although it was not until the latter half of the nineteenth century that a more formalized and regulated dental profession was established (Cohen, 1952). Dentistry at this time would have catered solely for the well‐to‐do social classes, however, and would have been out of reach of the poor. Members of the lower social classes would have relied on a “tooth‐puller” or resorted to folk cures such as “kiss[ing] the cross on a donkey's back,” “pull[ing] a jag out of a hedge‐hogs back with the sore tooth,” or “put[ting] a young frog into your mouth and squeeze[ing] on it with the sore tooth until it squeels [sic]” (Schools' Folklore Collection, 1937). We do not know if the laboring and poorer classes in nineteenth‐century Ireland would have been fully aware of the importance of keeping their teeth clean to prevent caries. The mechanisms used for cleaning teeth and how frequently these were employed are also uncertain. The evidence derived from the dentitions of those who perished in the Kilkenny Union Workhouse is suggestive that no dental treatment was available to these individuals beyond the possible extraction of teeth. The presence of high levels of calculus and caries, however, is suggestive that teeth were neither cleaned regularly nor efficiently.

**Figure 8:**
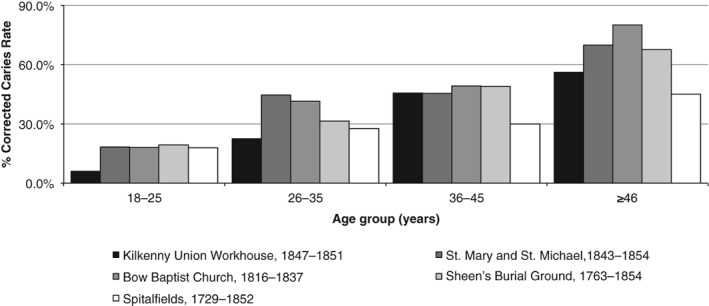
Comparison of corrected caries rates in adult dentitions between the population sample from the Kilkenny Union Workhouse and calculated rates from contemporaneous skeletal assemblages from London, England (based on frequency data in Hendersen, Miles, & Walker, 2013; Whittaker, 1993)

This study has shown that despite subsisting on a low‐cariogenic diet, tooth decay, and poor oral health was very much part of the life experience for the poor and laboring classes of nineteenth‐century Ireland. In comparative terms, however, the rates of tooth decay—when quantified using the caries correction factor—is similar (or even lower) than those of contemporaneous middle to upper class population groups from London, England (Figure 8), which may be an indication of the relatively low cariogenicity of the potato diet. It has not been possible to determine whether the anatomical pattern of caries differs between social groups in the nineteenth‐century in this study; however, as prevalence rates of carious lesions by tooth surfaces were not available from the published London data.

Periodontal disease also needs to be highlighted as a potentially very substantial health‐inhibiting condition in this population sample. Numerous clinical studies have focused on the link between periodontal disease and the morbidity of systemic diseases (Beukers, van der Heijden, van Wijk, & Loos, 2017; Elter, Champagne, Offenbacher, & Beck, 2004; Kim & Amar, 2006). A review article by Slots (2003) concluded that a link between periodontitis—which may harbor a reservoir of human viruses in inflamed gingiva—and systemic disease becomes particularly evident in immunocompromised individuals. A previous paleopathological study of the Kilkenny population sample has suggested that Vitamin C deficiency compromised the ability of these individuals to combat infectious diseases and greatly influenced their risk of death when entering the overcrowded workhouse institution. The mass mortality in this environment was mainly the result of infectious so‐called “famine diseases” such as typhus, typhoid fever and cholera (Geber & Murphy, 2012). The inclusion of periodontal disease, caused by lifestyle factors, as a potential contributor to the physical demise of a large proportion of the Irish population during the mid‐nineteenth century, further highlights the complexity and variability of the human experience of the Great Irish Famine.

As stated above, there is a known biological bias toward higher rates of caries and tooth loss in females compared to males due to hormonal factors (Lukacs & Largaespada, 2006). The relationship between parity and a decline in oral health has also been examined but studies have yielded contrasting results (e.g., Russell, Ickovics, & Yaffee, 2010; Scheutz, Baelum, Matee, & Mwangosi, 2002). Lukacs (2008) has argued for a link between an increase in fertility and elevated caries rates in bioarchaeological samples which are of interest when considering female oral health in nineteenth‐century Ireland. The significant upsurge in population growth that Ireland experienced from the mid‐seventeenth century onward was often explained as a result of earlier marriages and higher fertility rates in early historiography (Connell, 1950). Estimated crude birth rates available from census data from the 1830s onward, however, has indicated a steady decline in Irish fertility ratios in the decade before the Famine (Boyle & Ó Gráda, 1986). It is very possible the demographic changes that occurred in pre‐Famine Ireland not only resulted in social changes but also affected the oral health of its population.

In the modern world, oral health is strongly associated with socioeconomic factors and it seems probable that a similar link existed within nineteenth‐century societies. Poor oral health as a marker of poverty would also have impacted upon the quality of life of the affected individuals in a variety of manners. The types of food that could be consumed (if made available) would have been restricted due to the condition of the mouth. Affected individuals may have suffered from pain and halitosis and would have been at risk of developing infectious and systemic morbidities. Furthermore, they may also have suffered from psychosocial consequences arising from a negative self‐image as a consequence of tooth decay. The popularity of dental surgery and restoration work in the nineteenth century clearly reflects a consciousness of physical appearance (Cohen, 1952; Croll & Swanson, 2006; Miller, 1892; Nicholles, 1833). There is no reason to assume the poor who could not afford such treatment would have felt any differently.

## 5 CONCLUSIONS

This study has highlighted the methodological difficulties of assessing oral health in bioarchaeological samples; the rates of dental caries differ depending on whether they are quantified by crude prevalence by dentition, frequency by teeth, corrected caries rates or by using a t‐health index. Both males and females in this nineteenth‐century Irish population sample displayed poor oral health, even though they would have primarily relied on a potato and milk diet, which according to current clinical understanding is not particularly cariogenic. Both potato starch and lactose from milk can reduce the pH‐value of the oral cavity and, in the absence of oral hygiene practices, may have caused some tooth decay. Data derived from the early twentieth‐century Tristan da Cunha population that also subsisted largely on a potato diet, however, is indicative that low levels of diet‐related caries would have arisen. As such, diet alone cannot explain the high frequency of tooth decay in the dentitions of the Kilkenny Union Workhouse skeletons.

The explanation as to why this population sample exhibited such poor oral health is therefore, likely to relate to lifestyle factors and smoking in particular. The significant relationship between the presence of clay‐pipe facets, severity of periodontal disease, and occurrence of caries at, and below, the gum line are considered to have been due to habitual smoking. An age‐related pattern is suggestive that females started to smoke a pipe at an older age than males. It was rather surprising that even higher levels of caries were evident in contemporary middle and upper‐class populations in London, but this may be related to the fact that these groups would not have consumed a diet dominated by potatoes.

In the face of poverty and destitution, the horror of starvation and the mass death that occurred during the Great Famine, we might imagine that smoking would have been one of the comforts the poor could have enjoyed. Modern clinicians warn against the dangers of smoking for a myriad of reasons, and particularly its direct association with certain cancers and cardiovascular and pulmonary disease as a result of DNA damage, inflammation, and oxidative stress. Periodontitis and caries formation are recognized as potential side effects of smoking but the link is not considered to be definitive. The current study adds to the growing body of evidence that demonstrates that smoking is not only bad for your health; it is also bad for your teeth.

## Supporting information


**TABLE S1** Caries frequency (percentage, and absolute data below in square brackets) of permanent teeth, by age groups (years) and sex (M = male; F = female).

**TABLE S2** AMTL frequency (percentage, and absolute data below in square brackets) of permanent teeth, by age groups (years) and sex (M = male; F = female).
