# Supplementary material for: Dental markers of poverty: Biocultural deliberations on oral health of the poor in mid‐nineteenth‐century Ireland
Source: Am J Phys Anthropol. 2018 Oct 3;167(4):840–55. doi: 10.1002/ajpa.23717 (PMC6282970; doi:10.1002/ajpa.23717)
Supplement: Supplementary file 1 — TABLE S1 Caries frequency (percentage, and absolute data below in square brackets) of permanent teeth, by age groups (years) and sex (M = male; F = female). [file AJPA-167-840-s001.pdf]

TABLE S1 Caries frequency (percentage, and absolute data below in square brackets) of permanent teeth, by age groups (years) and sex (M = male; F = female).

Tooth (FDI)	18–25		26–35		36–45		≥46		Total	
	M	F	M	F	M	F	M	F	M	F
11+21	3.8<br>[1/26]	0.0<br>[0/25]	2.2<br>[1/46]	1.4<br>[1/74]	8.3<br>[7/84]	14.8<br>[9/61]	20.5<br>[8/39]	30.0<br>[3/10]	8.7<br>[17/195]	7.6<br>[13/170]
12+22	0.0<br>[0/28]	0.0<br>[0/24]	0.0<br>[0/48]	4.9<br>[4/81]	20.5<br>[15/73]	16.7<br>[11/66]	23.1<br>[9/39]	42.9<br>[3/7]	12.8<br>[24/188]	10.1<br>[18/178]
13+23	3.6<br>[1/28]	0.0<br>[0/30]	5.3<br>[3/57]	5.5<br>[5/91]	19.4<br>[19/98]	20.4<br>[19/93]	26.8<br>[15/56]	50.0<br>[8/16]	15.9<br>[38/239]	13.9<br>[32/230]
14+24	3.0<br>[1/33]	3.6<br>[1/28]	12.3<br>[7/57]	14.0<br>[12/86]	18.8<br>[18/96]	34.8<br>[31/89]	20.0<br>[10/50]	50.0<br>[3/6]	15.3<br>[36/236]	22.5<br>[47/209]
15+25	3.1<br>[1/32]	0.0<br>[0/29]	10.4<br>[7/67]	8.9<br>[8/90]	24.8<br>[25/101]	22.8<br>[18/79]	15.2<br>[7/46]	30.0<br>[3/10]	16.3<br>[40/246]	13.9<br>[29/208]
16+26	19.4<br>[6/31]	0.0<br>[0/29]	14.1<br>[9/64]	19.6<br>[18/92]	26.7<br>[24/90]	29.9<br>[23/77]	31.4<br>[11/35]	23.1<br>[3/13]	22.7<br>[50/220]	20.9<br>[44/211]
17+27	15.2<br>[5/33]	3.3<br>[1/30]	17.2<br>[10/58]	22.4<br>[19/85]	26.6<br>[21/79]	41.1<br>[23/56]	40.6<br>[13/32]	45.5<br>[5/11]	24.3<br>[49/202]	26.4<br>[48/182]
18+28	9.7<br>[3/31]	0.0<br>[0/20]	20.5<br>[8/39]	29.1<br>[16/55]	32.4<br>[22/68]	43.2<br>[16/37]	28.6<br>[6/21]	50.0<br>[2/4]	24.5<br>[39/159]	29.3<br>[34/116]
31+41	0.0<br>[0/15]	0.0<br>[0/12]	0.0<br>[0/26]	14.0<br>[6/43]	18.2<br>[8/44]	13.9<br>[5/36]	16.7<br>[3/18]	25.0<br>[2/8]	10.7<br>[11/103]	13.1<br>[13/99]
32+42	0.0<br>[0/29]	0.0<br>[0/25]	6.7<br>[4/60]	15.9<br>[14/88]	20.2<br>[20/99]	19.5<br>[16/82]	14.6<br>[6/41]	23.5<br>[4/17]	13.1<br>[30/229]	16.0<br>[34/212]
33+43	3.0<br>[1/33]	0.0<br>[0/29]	1.5<br>[1/68]	13.5<br>[13/96]	18.4<br>[21/114]	21.9<br>[21/96]	19.6<br>[10/51]	29.4<br>[5/17]	12.4<br>[33/266]	16.4<br>[39/238]
34+44	2.9<br>[1/34]	0.0<br>[0/28]	2.9<br>[2/70]	11.7<br>[12/103]	27.7<br>[36/130]	22.3<br>[23/103]	21.2<br>[11/52]	23.5<br>[4/17]	17.5<br>[50/286]	15.5<br>[39/251]
35+45	0.0<br>[0/33]	0.0<br>[0/28]	8.6<br>[6/70]	12.5<br>[13/104]	30.1<br>[37/123]	22.9<br>[22/96]	20.7<br>[12/58]	23.5<br>[4/17]	19.4<br>[55/284]	15.9<br>[39/245]
36+46	3.0<br>[1/33]	0.0<br>[0/31]	19.7<br>[14/71]	19.8<br>[19/96]	27.0<br>[31/115]	35.2<br>[31/88]	33.3<br>[17/51]	50.0<br>[6/12]	23.3<br>[63/270]	24.7<br>[56/227]
37+47	9.4<br>[3/32]	0.0<br>[0/28]	33.8<br>[24/71]	30.1<br>[25/83]	34.8<br>[32/92]	46.3<br>[31/67]	50.0<br>[20/40]	66.7<br>[4/6]	33.6<br>[79/235]	32.6<br>[60/184]
38+48	17.2<br>[5/29]	4.8<br>[1/21]	16.9<br>[11/65]	25.7<br>[18/70]	42.0<br>[37/88]	40.0<br>[24/60]	60.5<br>[26/43]	66.7<br>[4/6]	35.1<br>[79/225]	29.9<br>[47/157]
