# Supplementary material for: Dental markers of poverty: Biocultural deliberations on oral health of the poor in mid‐nineteenth‐century Ireland
Source: Am J Phys Anthropol. 2018 Oct 3;167(4):840–55. doi: 10.1002/ajpa.23717 (PMC6282970; doi:10.1002/ajpa.23717)
Supplement: Supplementary file 2 — TABLE S2 AMTL frequency (percentage, and absolute data below in square brackets) of permanent teeth, by age groups (years) and sex (M = male; F = female). [file AJPA-167-840-s002.pdf]

TABLE S2 AMTL frequency (percentage, and absolute data below in square brackets) of permanent teeth, by age groups (years) and sex (M = male; F = female).

Tooth (FDI)	18–25		26–35		36–45		≥46		Total	
	M	F	M	F	M	F	M	F	M	F
11+21	0.0 [0/33]	0.0 [0/28]	1.4 [1/70]	4.5 [5/110]	15.0 [22/147]	18.3 [22/120]	21.6 [16/74]	40.0 [10/25]	12.0 [39/324]	13.1 [37/283]
12+22	0.0 [0/31]	0.0 [0/29]	0.0 [0/71]	5.5 [6/109]	18.9 [28/148]	19.7 [24/122]	27.1 [19/70]	40.0 [10/25]	14.7 [47/320]	14.0 [40/285]
13+23	0.0 [0/34]	0.0 [0/30]	0.0 [0/72]	5.5 [6/110]	12.7 [19/150]	12.0 [15/125]	16.2 [12/74]	16.0 [4/25]	9.4 [31/330]	8.6 [25/290]
14+24	0.0 [0/34]	3.4 [1/29]	1.4 [1/73]	9.2 [10/109]	14.5 [21/145]	13.2 [16/121]	14.1 [10/71]	34.8 [8/23]	9.9 [32/323]	12.4 [35/282]
15+25	0.0 [0/34]	3.3 [1/30]	0.0 [0/74]	8.3 [9/108]	16.9 [24/142]	17.2 [20/116]	24.2 [16/66]	43.5 [10/23]	12.7 [40/316]	14.4 [40/277]
16+26	6.1 [2/33]	6.3 [2/32]	6.8 [5/73]	12.4 [13/105]	25.2 [34/135]	30.4 [34/112]	35.0 [21/60]	50.0 [9/18]	20.6 [62/301]	21.7 [58/267]
17+27	0.0 [0/33]	3.2 [1/31]	11.3 [8/71]	12.2 [12/98]	30.6 [38/124]	41.1 [44/107]	42.3 [22/52]	22.2 [2/9]	24.3 [68/280]	24.1 [59/245]
18+28	0.0 [0/33]	0.0 [0/21]	2.3 [1/43]	13.4 [9/67]	25.2 [27/107]	40.0 [36/90]	37.2 [16/43]	83.3 [5/6]	19.5 [44/226]	27.2 [50/184]
31+41	0.0 [0/33]	6.5 [2/31]	10.4 [8/77]	10.7 [13/122]	22.4 [36/161]	23.8 [30/126]	30.4 [24/79]	35.9 [14/39]	19.4 [68/350]	18.6 [59/318]
32+42	0.0 [0/34]	0.0 [0/32]	3.8 [3/78]	7.3 [9/123]	13.5 [22/163]	12.0 [16/133]	19.5 [16/82]	33.3 [12/36]	11.5 [41/357]	11.4 [37/324]
33+43	0.0 [0/34]	0.0 [0/32]	0.0 [0/78]	4.1 [5/121]	7.7 [13/169]	8.0 [11/137]	10.8 [9/83]	27.0 [10/37]	6.0 [22/364]	8.0 [26/327]
34+44	0.0 [0/34]	0.0 [0/32]	0.0 [0/78]	5.9 [7/119]	9.1 [15/165]	14.0 [19/136]	14.1 [12/85]	31.6 [12/38]	7.5 [27/362]	11.7 [38/325]
35+45	0.0 [0/34]	6.3 [2/32]	3.7 [3/81]	5.0 [6/119]	15.0 [24/160]	21.8 [29/133]	20.0 [17/85]	44.7 [17/38]	12.2 [44/360]	16.8 [54/322]
36+46	3.0 [1/33]	9.4 [3/32]	8.8 [7/80]	28.9 [35/121]	32.7 [54/165]	45.5 [61/134]	45.9 [39/85]	78.9 [30/38]	27.8 [101/363]	39.7 [129/325]
37+47	0.0 [0/34]	9.4 [3/32]	6.2 [5/81]	28.7 [35/122]	37.7 [61/162]	48.9 [65/133]	47.0 [39/83]	84.6 [33/39]	29.2 [105/360]	41.7 [136/326]
38+48	0.0 [0/25]	17.6 [3/17]	5.9 [4/68]	31.3 [30/96]	31.9 [43/135]	45.8 [55/120]	41.3 [31/75]	73.0 [27/37]	25.7 [78/303]	42.6 [115/270]
